# The efficacy of a blended intervention to improve physical activity and protein intake for optimal physical recovery after oncological gastrointestinal and lung cancer surgery, the Optimal Physical Recovery After Hospitalization (OPRAH) trial: study protocol for a randomized controlled multicenter trial

**DOI:** 10.1186/s13063-023-07705-2

**Published:** 2023-11-27

**Authors:** Marijke de Leeuwerk, Vincent de Groot, Suzanne ten Dam, Hinke Kruizenga, Peter Weijs, Edwin Geleijn, Marike van der Leeden, Marike van der Schaaf, on behalf of the OPRAH consortium, Chris Dickhoff, Marc G. Besselink, Jurriaan B. Tuynman, Mark I. van Berge Henegouwen, Joris I. Erdmann, Rosalie J. Huijsmans, Hidde P. van der Ploeg, Anne M. Eskes, Mirjam A. G. M. Pijnappels, Liesbeth Schuijs van Leeuwen, Anke B. Smits, Jasmijn van Dijk, Eva Grimbergen

**Affiliations:** 1 Amsterdam UMC, Location Vrije Universiteit Amsterdam, Rehabilitation Medicine, De Boelelaan 1117, 1081 HV Amsterdam, The Netherlands; 2 Amsterdam Movement Sciences, Ageing & Vitality, Amsterdam, The Netherlands; 3 Amsterdam Movement Sciences, Rehabilitation & Development, Amsterdam, The Netherlands; 4 Amsterdam UMC, Location Vrije Universiteit Amsterdam, Nutrition and Dietetics, De Boelelaan 1117, Amsterdam, The Netherlands; 5 Center of Expertise Urban Vitality, Amsterdam University of Applied Sciences, Amsterdam, the Netherlands; 6 Amsterdam UMC, Location University of Amsterdam, Rehabilitation Medicine, Meibergdreef 9, Amsterdam, The Netherlands; 7 Department of Cardio-thoracic Surgery, Amsterdam UMC, location Vrije Universiteit Amsterdam, Amsterdam, The Netherlands; 8 Department of Surgery, Amsterdam UMC, location Vrije Universiteit Amsterdam, Amsterdam, The Netherlands; 9 Department of Public and Occupational Health, Amsterdam UMC, location Vrije Universiteit Amsterdam, Amsterdam, The Netherlands; 10 Department of Human Movement Sciences, Vrije Universiteit Amsterdam, Amsterdam, The Netherlands; 11 Department of Surgery, St. Antonius Hospital, Nieuwegein, The Netherlands; 12 Department of Paramedical treatment and Rehabilitation, St. Antonius Hospital, Nieuwegein, The Netherlands; 13 Department of Nutrition and Dietetics, St. Antonius Hospital, Nieuwegein, The Netherlands

**Keywords:** Cancer, Surgery, Supportive care, Physical functioning, Physical activity, Protein intake

## Abstract

**Background:**

Improving physical activity, especially in combination with optimizing protein intake, after surgery has a potential positive effect on recovery of physical functioning in patients after gastrointestinal and lung cancer surgery. The aim of this randomized controlled trial is to evaluate the efficacy of a blended intervention to improve physical activity and protein intake after hospital discharge on recovery of physical functioning in these patients.

**Methods:**

In this multicenter single-blinded randomized controlled trial, 161 adult patients scheduled for elective gastrointestinal or lung cancer surgery will be randomly assigned to the intervention or control group. The purpose of the Optimal Physical Recovery After Hospitalization (OPRAH) intervention is to encourage self-management of patients in their functional recovery, by using a smartphone application and corresponding accelerometer in combination with coaching by a physiotherapist and dietician during three months after hospital discharge. Study outcomes will be measured prior to surgery (baseline) and one, four, eight, and twelve weeks and six months after hospital discharge. The primary outcome is recovery in physical functioning six months after surgery, and the most important secondary outcome is physical activity. Other outcomes include lean body mass, muscle mass, protein intake, symptoms, physical performance, self-reported limitations in activities and participation, self-efficacy, hospital readmissions and adverse events.

**Discussion:**

The results of this study will demonstrate whether a blended intervention to support patients increasing their level of physical activity and protein intake after hospital discharge improves recovery in physical functioning in patients after gastrointestinal and lung cancer surgery.

**Trial registration:**

The trial has been registered at the International Clinical Trials Registry Platform at 14–10-2021 with registration number NL9793. Trial registration data are presented in Table 1.

**Supplementary Information:**

The online version contains supplementary material available at 10.1186/s13063-023-07705-2.

## Introduction

Major surgical procedures for gastrointestinal (GI) and lung cancer frequently result in significant loss of muscle mass, caused by increased catabolism due to the surgical stress response. This has major implications for postoperative physical function and has been associated with postoperative morbidity, mortality and quality of life [1–6]. Adequate physical activity and nutrition are important to prevent loss of muscle mass [7–10]. A combination of both is even more important, as adequate protein intake is needed to optimally benefit from the physical training stimuli [11]. This has also been reflected in the results from a systematic review showing that a combination of adequate protein intake and sufficient physical activity facilitates muscle gain in sarcopenia [12]. Therefore, to minimize the postoperative loss of muscle mass and restore physical function, it is important for patients with cancer undergoing surgery to maintain or enhance their physical activity and nutritional status in the postoperative phase (Table 1).

**Table 1:** Trial registration data set

Data category	Information
Primary registry and trial identifying number	International Clinical Trials Registry Platform NL9793
Date of registration in primary registry	14 October, 2021
Secondary identifying numbers	NL78840.029.21
Source(s) of monetary or material support	Amsterdam UMC, location VUmc, department of Rehabilitation
Primary sponsor	Amsterdam UMC, location VUmc, department of Rehabilitation
Secondary sponsor(s)	Amsterdam Movement Sciences Institute
Contact for public queries	MdL, m.e.deleeuwerk@amsteramumc.nl
Contact for scientific queries	MdL, m.e.deleeuwerk@amsteramumc.nl Amsterdam UMC, location VUmc, department of Rehabilitation, Amsterdam, The Netherlands
Public title	Optimal Physical Recovery After Hospitalization (OPRAH study)
Scientific title	The efficacy of a blended intervention to improve physical activity and protein intake for optimal physical recovery after oncological gastrointestinal and lung cancer surgery: study protocol for a randomized controlled multicenter trial
Countries of recruitment	The Netherlands
Health condition(s) or problem(s) studied	Rehabilitation after oncological surgery
Intervention(s)	Intervention: Smartphone application and corresponding accelerometer in combination with coaching by a physiotherapist and dietician during three months after hospital discharge
	Control: Usual care
Key inclusion and exclusion criteria	Ages eligible for study: ≥ 18 years Sexes eligible for study: both Accepts healthy volunteers: no
	Inclusion criteria: adult patient (≥ 18 years), scheduled for curative intent surgery for gastrointestinal cancer, including esophageal and stomach cancer (upper GI), colorectal and hepato-pancreato-biliary (HPB) cancer, or lung cancer with a planned hospital stay of ≥ 2 nights, able to fill in online questionnaires in Dutch and give informed consent
	Exclusion criteria: pulmonary wedge resection, surgery with open/close procedure, having no access to a mobile device compatible for applications, less than 5 days between inclusion and surgery, patients who are wheelchair dependent, a Mini-Mental State Examination (MMSE) ≤ 24 and already participating in a conflicting study
Study type	Multicenter randomized controlled intervention trial with allocation at level of the individual
	Allocation: randomized
	Primary purpose: treatment
	Phase III trial
Date of first enrolment	June 2022
Target sample size	161
Recruitment status	Recruiting
Primary outcome(s)	Recovery in physical functioning six months after hospital discharge
Key secondary outcomes	Physical activity, lean body mass, pain, fatigue, muscle mass, protein intake, physical performance, patient-specific activity limitations, self-efficacy, participation in social roles and activities, generic quality of life, global perceived effect, hospital readmission and adverse events

However, patients often experience barriers to being physically active after surgery, e.g. due to physical symptoms, such as pain and fatigue, and lack of motivation or social support [13–16]. In addition, previous studies found that many surgical patients were unable to meet their protein requirements after surgery despite the advices of a dietician, e.g. due to a loss of appetite or feelings of worry [17]. Patients emphasize the need for more supportive care interventions after discharge to facilitate return to normal activities after cancer surgery [18, 19]. Therefore, additional support in promoting physical activity and protein intake is needed to improve recovery of physical functioning after surgery in these patients [20].

Self-monitoring of physical activity with the use of accelerometers is an often used strategy to increase physical activity in patients, by giving patients insight in their daily physical activity level [21]. A recent meta-analysis showed that interventions combining accelerometers with feedback using different behavioral change techniques (BCTs) and coaching by a health care professional are more effective in increasing physical activity than the use of an accelerometer alone [22]. In this study, it is suggested that this is due to the fact that incorporating coaching by a health professional to the intervention gives the opportunity to provide targeted advice and interventions for a specific population group with a more personal touch. Also, more BCTs can be used when a health care professional is involved, e.g. problem solving, social reward. In addition, findings of a review on patients with a colorectal adenoma indicate that behavioral interventions can encourage these patients to improve their diet [23]. The effect of a combined intervention, using eHealth and remote coaching by a dietician and physiotherapist in patients after GI or lung cancer is unknown.

We therefore developed a blended intervention to support patients in increasing their level of physical activity and protein intake after hospital discharge: The Optimal Physical Recovery After Hospitalization (OPRAH) intervention. The purpose of the OPRAH intervention is to encourage self-management of patients in their recovery in physical functioning, by using a smartphone application and corresponding accelerometer in combination with coaching by a physiotherapist and dietician. To investigate the potential effect of providing ongoing support on physical activity and protein intake after hospital discharge on recovery in physical functioning, the intervention will be compared to usual care. Therefore, the aim of this randomized controlled multicenter trial is to investigate the effectiveness of the OPRAH intervention on recovery of physical functioning, compared to usual care, in patients who have undergone elective GI and lung cancer surgery.

### Objective and hypothesis

*Objective:* to evaluate the efficacy of the OPRAH intervention on recovery of physical functioning (compared with usual care) in patients who have undergone elective GI and lung cancer surgery.

*Hypothesis*: The aim of the intervention is to encourage self-management of patients by the use of self-monitoring on physical activity and protein intake. Patients will also be monitored in their recovery of physical activity and protein intake by a physiotherapist and dietician. If this recovery stagnates and goals are not achieved, the physiotherapist or dietician can contact the patient to identify barriers in their recovery. The use of multiple BCTs can help to reduce or eliminate these barriers and to increase the patient’s level of physical activity and protein intake. Higher levels of physical activity and achieving protein requirements are expected to have a positive effect on the recovery in physical functioning after discharge. Therefore, it is hypothesized that patients in the intervention group will have a faster and better recovery in physical functioning during the first 6 months after hospital discharge compared to patients receiving usual care.

## Methods

### Study design

**Fig. 1:**
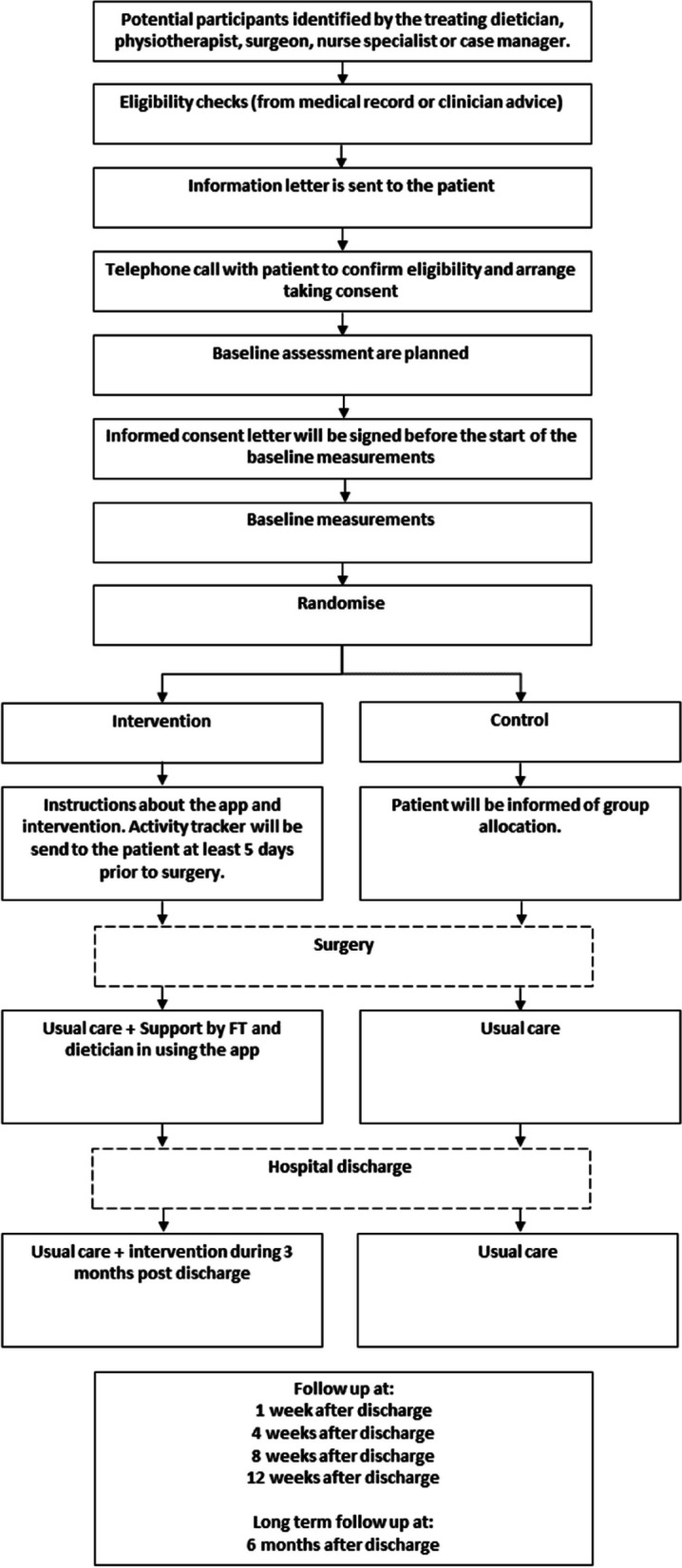
Flow chart

**Fig. 2:**
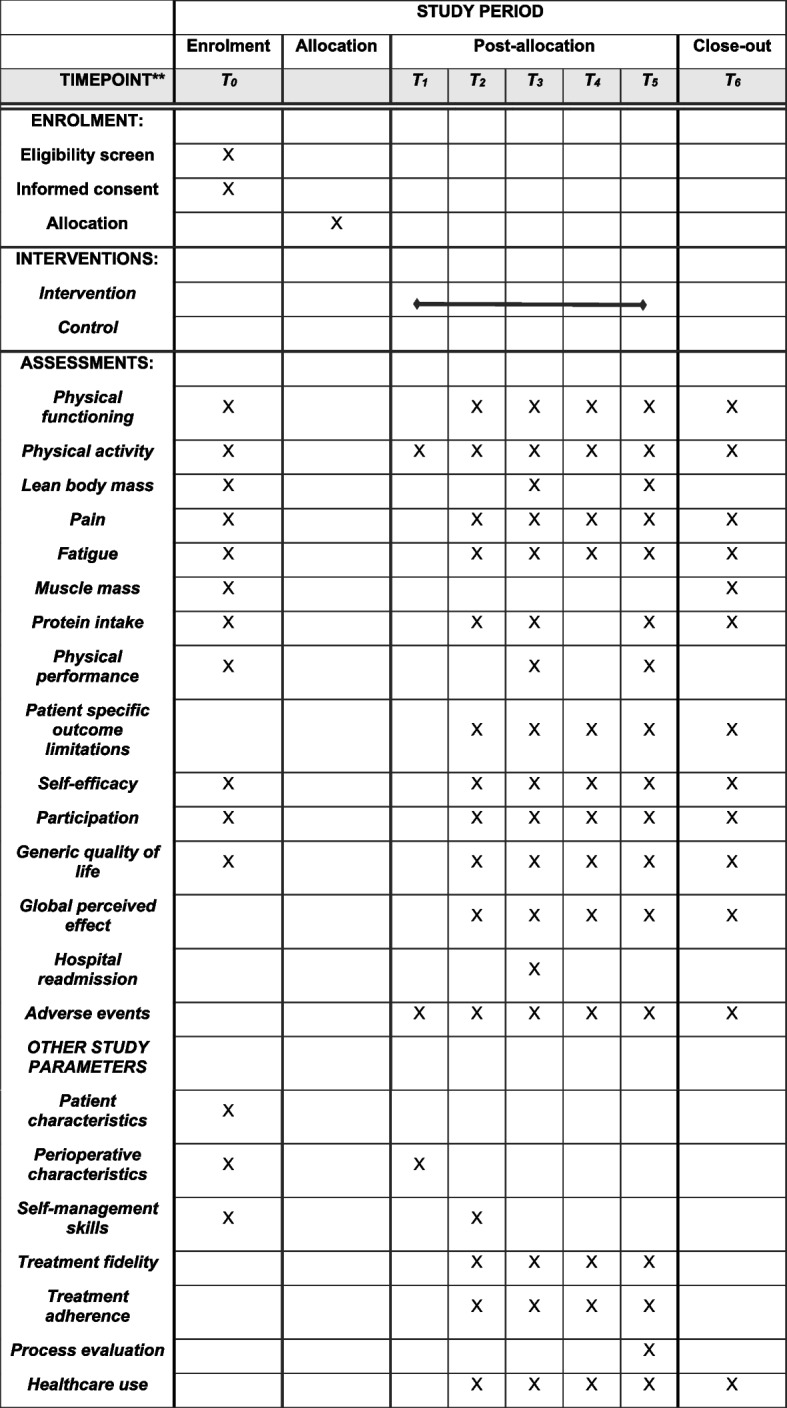
SPIRIT schedule of enrolment, intervention, and assessments

The proposed study is a multicenter, single-blinded two-arm randomized controlled study comparing a blended intervention delivered alongside usual care, with a control arm (usual care) in patients after hospital discharge who have undergone GI or lung cancer surgery. Baseline measurements (*T*_0_) will be conducted prior to surgery and follow-up measurements take place at hospital discharge (*T*_1_) and 1 (*T*_2_), 4 (*T*_3_), 8 (*T*_4_) and 12 (*T*_5_) weeks and 6 months (*T*_6_) after hospital discharge. The trial will be conducted at two hospitals in the Netherlands: Amsterdam UMC, location VUmc and St. Antonius, location Nieuwegein. The OPRAH trial has been designed in accordance with the Consolidated Standards of Reporting Trials (CONSORT) statement [24]. The Standard Protocol Items Recommendations for Interventional Trial (SPIRIT) checklist is provided as additional file. See Fig. 1 for the flowchart of the study and Fig. 2 for the SPIRIT schedule of enrollment, intervention and assessments.

### Participants

#### Eligibility criteria

Patients are eligible to participate when scheduled for curative intent surgery for gastrointestinal cancer, including esophageal and stomach cancer (upper GI), colorectal and hepato-pancreato-biliary (HPB) cancer, or lung cancer with a planned hospital stay of ≥ 2 nights, are aged 18 years or older, and whether they are able to fill in online questionnaires in Dutch and give informed consent.

Exclusion criteria are the following: pulmonary wedge resection, surgery with open/close procedure, having no access to a mobile device compatible for applications, less than 5 days between inclusion and surgery, patients who are wheelchair dependent, a Mini-Mental State Examination (MMSE) ≤ 24 and already participating in a conflicting study.

#### Recruitment

Potentially eligible patients will be informed about the study by the treating dietician, physiotherapist, nurse specialist or case-manager during a preoperative consultation. When the patient is interested in participation, the researcher will contact the patient after 24 h to further explain the study procedures and to answer questions of the patient. If the patient is eligible and willing to participate, the informed consent letter will be signed before the start of the baseline measurements. During the informed consent procedure, participants are also asked to confirm if their data may be used to support other research in the future. Participants will also indicate whether they would be willing to be contacted about future-related research and if they give consent to the making, use and retention of audio recordings of conversations with the dietitian and physiotherapist. In case there are doubts about the patients’ cognition, the MMSE will be administered before final inclusion. The patient receives a copy of the signed informed consent. The recruitment period is 18 months, with a target of approximately 10 included participants per month.

#### Randomization and blinding

After the baseline measurements, patients will be randomly assigned to the control or intervention group with a 1:1 allocation ratio using the randomization tool of Castor Electronic Data Capture (EDC) [25]. Randomization will be stratified per center by type of surgery (lung, HPB, upper GI, colorectal) and ASA score (1–2 or ≥ 3). The randomization tool of Castor ensures concealment of allocation. The researcher informs the participant by e-mail which group he has been assigned to. Assessments will be conducted by a blinded research assistant. Neither the patient nor the therapist will be blinded.

#### Sample size

For the present sample size analysis, a conservative estimate of 0.40 as the between-group effect size on the outcome physical functioning is used. This estimate is based on reported effect sizes on patient-reported outcomes of physical functioning in other studies using technology and coaching on physical activity [26, 27]. Based on alpha = 0.05, power (1 − *β*) = 0.80, a two-sided test for repeated measures with an expected within-subject correlation coefficient of 0.6 and 5 follow-up measurements the minimum number of 67 subjects per group is required, with a total sample size of 134 (see Formula 1). Allowing for a drop-out rate of 20% during the study, this study should include 161 patients.

The most important secondary outcome of this study is objectively measured physical activity. Therefore, a sample size calculation was also made on this outcome measure. Based on the effect sizes found in our recent systematic review and meta-analysis of interventions using activity trackers in patients during or after inpatient stay on the outcome physical activity, an effect size on physical activity of 0.50 is expected [22]. Based on alpha = 0.05, power (1 − beta) = 0.80 and a two-sided test the minimum number of subjects required is *n* = 128 (64 in each group). Allowing a drop-out rate of 20% during the study, a total of 154 patients should be included.

Formula 1:





### Intervention

The intervention is described according to the template for intervention description and replication (TIDIER) checklist (See Supplementary File 1). The main purpose of the OPRAH intervention is to facilitate faster and better recovery in physical functioning by stimulating patients’ self-management regarding their level of physical activity and protein intake after hospital discharge.

#### Development of the intervention

The Medical Research Council (MRC) framework for the development and evaluation of complex intervention was used [28]. Supplementary File 3 shows the stages of the MRC framework alongside with our activities of the development process and the activities that are described in this paper. The intervention development process was guided by findings from a feasibility study [29], systematic review of literature on the effectiveness of intervention components [22], a literature search about barriers and facilitators to the targeted behavior and expert meetings with researchers and health professionals (OPRAH consortium, consisting of physiotherapists, dieticians, surgeons, researchers and a specialist in behavioral change). The behavioral change wheel was used as theoretical underpinning of the intervention [30]; with the use of this theory, we have been able to substantiate how the intervention causes change, what the active ingredients of the intervention are and how they can exert their effect. A feasibility study was conducted to evaluate the practical effectiveness.

The basis of the intervention was an existing app with a self-monitoring function of physical activity, which had been investigated in the postoperative period of oncological patients through a feasibility study [29]. Self-monitoring appeared to be feasible in this population. However, some patients emphasized the need for more support in addition to self-monitoring. This finding was strengthened by the results from our systematic review, because interventions with activity trackers in combination with coaching by a health professional seemed to be more effective in increasing physical activity during and after hospitalization [22]. In addition, the use of more behavioral change techniques (BCT’s) within the intervention was also suggested to be more effective.

Because of the important synergy between protein intake and physical activity after major oncological surgery, the app has been expanded with a self-monitoring tool for protein intake. By conducting a comprehensive literature search, barriers and facilitators to the targeted behaviors, improving physical activity and protein intake, were identified. Based on the behavioral change wheel and with input from the OPRAH consortium, a total of 15 behavioral change techniques, following the BCT taxonomy of Michie et al. [31], were identified and linked to a mode of delivery in order to target the desired behavior. (See Table 2) In Supplementary File 4, the BCTs are linked to the Capability, Opportunity, Motivation and Behavior (COM-B) model [30], intervention functions and mode of delivery. In order to improve the motivation of patients for behavioral change, motivational interviewing (MI) and shared decision making (SDM) was incorporated in the intervention. Based on initial feedback from the OPRAH consortium, the intervention was refined in preparation for evaluation.

**Table 2:** Intervention components based on the BCT taxonomy (v1) of 93 hierarchically cluster techniques from Michie et al. [31]

Behavioral change technique	Description
**1.1 Goal setting (behavior)**	Patients are able to set goals on the amount of physical activity per day. Patients will be supported by the physiotherapist to set realistic goals Goals on requirements of protein intake will be set based on advice of the dietician
**1.2 Problem solving**	The physiotherapist/dietician analysis factors influencing the behavior and select strategies for overcoming barriers/increasing facilitator to perform behavior
**1.4 Action planning**	Patients are able to set in-app tasks. Patients will be encouraged by the physiotherapist/dietician to plan the performance (when, what time etc.)
**1.5 Review behavioral goals**	The physiotherapist/dietician will review the behavioral goals and consider modifying goals based on their achievement
**1.6 Discrepancy between current behavior and goal**	Visual in-app presentation of behavior and targeted goals
**2.2 Feedback on behavior**	The amount of minutes patients have to be active to achieve their goal is presented in the app. The number of points remaining to achieve protein requirements is shown In addition, the physiotherapist/dietician will give the patient feedback about their activity/intake
**2.3 Self-monitoring of behavior**	Patients are able to monitor their daily level of physical activity and protein intake via the app
**3.1 Social support**	Patients are able to request contact with the physiotherapist/dietician via the app The physiotherapist/dietician will contact the patient (how often is determined in consultation with the patient)
**4.1 Instructions on how to perform the behavior**	Tailored in-app information and personalized instructions by physiotherapist/dietician
**5.1 Instructions about health consequences**	Tailored in-app information and personalized instructions by physiotherapist/dietician
**7.1 Prompts/cues**	Patients can have the opportunity to receive in-app reminders to reach their daily goal
**8.7 Graded tasks**	The physiotherapists stimulate patients to set easy-to-perform tasks
**10.4 Social reward**	Physiotherapist/dietician reward the patients if there has been effort in performing the behavior
**10.5 Social incentive**	Patients receive in-app rewards if they achieved their goal
**12.5 Adding objects to the environment**	Wearing the PAM sensor

#### Coaching by health professionals

Coaching is an important part of the intervention, as the use of self-monitoring has proven to be more effective when combined with coaching by a healthcare professional and is considered important to improve the synergy and collaboration between physiotherapy and dietetics. Through the use of coaching, the intervention can be tailored to the clinical status of the patient. In addition, potential barriers to the desired behavior can be identified and, if they are within the scope of physiotherapist and dietician, addressed in collaboration with the patient. To support the physiotherapists and dieticians in coaching, the choice has been made to use motivational interviewing (MI) and shared decision making (SDM). MI increases patient autonomy, enhances intrinsic motivation and supports the patient’s self-efficacy all of which contributes to increasing the patient’s self-management [32]. A recent review indicated that MI is a powerful intervention in combination with self-monitoring using activity trackers to improve autonomous motivation and to reduce a-motivation for physical activity [33]. Furthermore, it was indicated that the delivery of the intervention can vary from telephone to real life coaching and can still be effective in impacting motivation, regardless of the delivery method [33]. SDM provides an opportunity to integrate evidence and patient preferences into a health-related decision [34, 35]. The physiotherapist and dieticians involved in this study have received a training about MI and SDM prior to the start of the study. The main purpose of this 3-day training course was to teach strategies according to the principles of MI to encourage patients to adopt healthy behavior, especially focused on physical activity and protein intake. In the first session, attention was paid to reflective listening; i.e. listening carefully to what the patient says and giving it back to the patient in different words in order to create understanding and clarity. Next, it was discussed what ambivalence is and how it can be recognized. Ambivalence means being pulled back and forth between the disadvantages and advantages of the current situation and the new situation. In the second session, the recognition of ambivalence was continued and conversation techniques were applied in order to guide the patient towards healthy behavior. During the last training day, all techniques were practiced with the help of a trained actor. Between the training days, the physiotherapists and dieticians applied the learned techniques in practice and reflected on this in duo sessions. To keep their knowledge and skills up to date during the study period, peer review meetings will be organized.

#### E-health technology

**Fig. 3:**
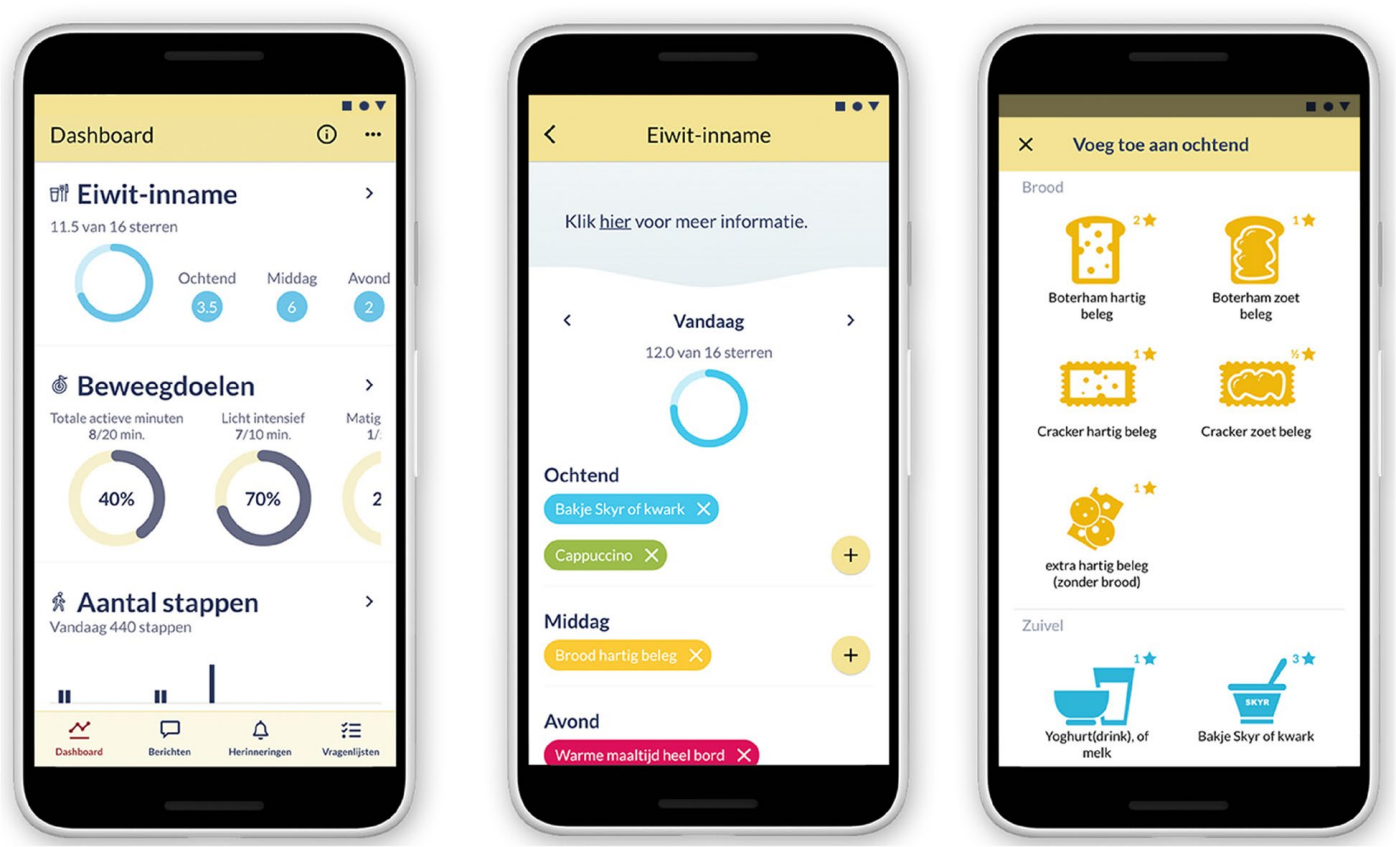
Screenshots of the Atris app

The Atris software (Peercode B.V. Geldermalsen, the Netherlands) is the investigational software used in the intervention. In combination with the ankle-worn PAM AM400 three-axis accelerometer (PAM B.V. Doorwerth, the Netherlands), the Atris software is used for self-monitoring of physical activity and protein intake. The Atris software consists of an Atris app for patients, Atris backend website for health professionals and the Atris (triggers) software. The Atris app allows patients to self-monitor their daily physical activity and protein intake through wearing the PAM and tracking daily protein intake by using a simple in-app self-registration system. See Fig. 3 for screenshots of the app and Supplementary File 2 for a subscript of the screenshots. Physical activity is represented in active minutes per day, with a distinction in low (1.4–2.99 metabolic equivalents of energy expenditure (METs)), moderate (3–7 METs), and vigorous activity (> 7 METs). The protein intake is represented in stars (★), where 1 star represents approximately 5 g of protein. The Atris app provides feedback on progress related to their goals. In addition, patients can ask questions through the app’s chat feature and receive response and information by the physiotherapist or dietician. Via the Atris backend website, the physiotherapist and dietician can interactively view patient data, send messages to the patient, and can access Atris (triggers) through the patient monitor. The software Atris (triggers) enables personalized goal setting and threshold values per patient.

#### Intervention description and procedures

One week prior to planned surgery, patients receive the PAM and will receive access to and get instructions about the Atris app to get familiarized with the application. Patients are asked to wear the PAM 24 h a day in a strap around the ankle, from at least 5 days prior to surgery until 3 months after surgery. During hospitalization, the treating physiotherapist and dietician guide the patients in the use of the app during their standard consultations. As the day of hospital discharge approaches, the patient will be supported by the physiotherapists and dietician using the SDM process to set goals on active minutes and protein intake for after discharge. After discharge to home, patients are coached remotely (by telephone and chat) by a physiotherapist and dietician about physical activity and protein intake during 3 months after discharge. Through a chat function in the app, patients can ask questions to the physiotherapist or dietician, the physiotherapist and dietician can also send information through the chat. The ultimate goal for physical activity is to return to pre-surgery level of physical activity. The ultimate goal for protein intake is to achieve the personal daily requirements. If the recovery stagnates and goals are not achieved, the physiotherapist or dietician will contact the patient to identify barriers in their recovery. The sub-goals and the degree of coaching will be tailored using a SDM process to the personal needs and preferences of the patient. To support patients’ self-management, MI techniques will be applied during the coaching sessions with the physiotherapist and dietician by telephone and chat [32].

#### Criteria for discontinuing the intervention

Patients may discontinue the intervention in the following cases:

- Completion of the intervention period: Patients may discontinue the intervention once they have completed the predetermined duration of the OPRAH intervention (3 months after hospital discharge).
- Adverse effects or complications: If patients experience any adverse effects or complications directly related to the intervention, it may be necessary to discontinue their participation for safety reasons.
- Lack of adherence: If patients consistently fail to comply with the requirements or recommendations of the intervention, discontinuation will be considered. This can include non-engagement with wearing the PAM sensor or registration of their protein intake.
- Patient’s request or withdrawal: Patients have the right to choose whether they want to continue or discontinue the intervention. If a patient decides to withdraw from the program, their participation will be discontinued.

### Usual care

Both participants in the intervention and the control group receive usual care.

During hospitalization, patients are treated according to the Early Recovery After Surgery (ERAS) protocol [36]. This includes early mobilization and (nutritional) intake supported by the entire (para)medical team. In the daily consultations by the medical doctor and nursing staff, attention is paid to the improvement of mobilization and intake. The amount of consultation by the physiotherapist and dietician is determined based on the clinical assessment of the physiotherapist and dietician.

After hospital discharge, there is no usual physiotherapy care. The physiotherapist may advise the patient to continue physiotherapy in primary care, based on the clinical assessment of the physiotherapist. The usual care of the dietician differs between patient groups. In Amsterdam UMC, the dietician standard schedules postoperative consultations with patients after esophagus-, stomach-, pancreas-, biliair and Hypertherme Intraperitoneale Chemotherapy (HIPEC) cancer surgery at 2 and 4 weeks and 3 and 6 months after discharge. At St. Antonius, the dietician only schedules standard postoperative consultations at 2 weeks after discharge for patients after pancreas surgery. When necessary, more consultations can be planned. Patients after hepatic, colorectal (excl. HIPEC) or lung cancer surgery receive postoperative consultations by the dietician only on indication or the dietician may advise the patient to continue dietetic treatment in primary care. All participants are permitted to engage any form of (para)medical care during the study period.

### Adverse event reporting

#### Adverse events (AEs)

Adverse events are defined as any undesirable experience occurring to a subject during the study, whether or not considered related to experimental intervention. All adverse events with a direct or possible link to the OPRAH trial (e.g. AEs occurring during intervention-related activities) reported spontaneously by the subject or observed by the investigator or his staff will be recorded.

#### Serious adverse events (SAE)

This study includes patients undergoing oncological lung or GI surgery. These types of surgery are associated with a certain risk of postoperative complications. Our intervention starts after hospital discharge; therefore, all complications during hospitalization will not be reported as SAE. We expect that our, low-risk, post-discharge intervention will not have any negative influence on the occurrence complications after discharge. Therefore, all complications after discharge which are unmistakably caused by the surgery and/or medical treatment will not be reported as SAE. When there are doubts about the relation between the intervention and the occurrence of an SAE, these SAEs will be discussed with the surgeons involved in this study per patient group. If, after discussion, there is still any doubt about the relation between the intervention and the occurrence of an SAE, the SAE will be reported.

The participating hospitals will report SAEs within 24 h to the sponsor. The sponsor will report the SAEs to the accredited medical ethical committee that approved the protocol, within 7 days of first knowledge for SAEs that result in death or are life threatening followed by a period of maximum of 8 days to complete the initial preliminary report. All other SAEs will be reported within a period of maximum 15 days after the sponsor has first knowledge of the serious adverse events.

### Outcomes

The primary outcome is recovery in physical functioning 6 months after hospital discharge and the most important secondary outcome is physical activity. Other secondary outcomes are lean body mass, pain, fatigue, muscle mass, protein and energy intake, physical performance, patient-specific activity limitations, self-efficacy, participation in social roles and activities, generic quality of life, global perceived effect, hospital readmission and adverse events. See Table 3 for a detailed description of the outcome measures with the corresponding follow-up time points.

**Table 3:** Outcome measures

Construct	Measure	Abbrev	Description	Time points
***Primary outcome***				
Physical functioning	Computer Adaptive Testing (CAT) Dutch-Flemish Patient-Reported Outcome Measure Information System for Physical Functioning	CAT PROMIS-PF	The CAT PROMIS-PF is a digital questionnaire [37]. A CAT is a computer-administered measure in which a computer algorithm is used to select successive items based on responses to previous items. Using a 5-point Likert scale the questionnaire reflects the participant’ functioning in the past 7 days. The items cover a wide range of activities, from activities of daily living to more complex activities [38]. The CAT PROMIS-PF has sufficient psychometric properties in measuring the level of physical function of physiotherapy patients [39] and has been shown to be reliable and sensitive to change in surgical patients [40, 41].	T0, T2, T3, T4, T5, T6
***Most important secondary outcome***				
Physical activity	ActivPAL™		The ActivPAL (PAL Technologies Ltd., Glasgow, UK) is a thigh-worn tri-axial accelerometer and uses proprietary analysis algorithms to determine posture (sedentary time, upright time) and stepping (stepping time and steps). The Activpal will be affixed to the skin with hydrogel pads on the thigh. The ActivPAL is one of the most frequently used activity trackers in clinical research and has been shown to be a valid measure of posture and stepping [42–44]. Patients in both the intervention and control group will wear the ActivPAL for 5 days	T0, T1, T3, T5, T6
***Other secondary outcomes***				
Lean body mass	Bioelectrical Impedance Analysis	BIA	A single frequency bio electric impedance meter (50 kHz) will be used for the BIA. These impedance meters uses 4 electrodes to measures the impedance, resistance, reactance and phase angle. A BIA is a non-invasive validated method to assess body composition, fat-free mass (FFM) and appendicular skeletal muscle mass (ASSM) patients with cancer [45]. FFM is one of the factors the dietician uses to determine patients’ personal protein requirements. FFM will be measured with the formula of Kyle [46]	T0, T3, T5
Pain	Numeric Pain Rating Scale	NPRS	The NPRS is a measure of subjective intensity of pain in adult patients. The 11-point numeric scale ranges from ‘0’ (no pain) to ‘10’ (worst pain imaginable). The NPRS is a valid, reliable and usual tool to measure pain in postoperative patients [47, 48].	T0, T2, T3, T4, T5, T6
Fatigue	Dutch-Flemish Patient-Reported Outcome Measure Information System for Fatigue – Short Form	PROMIS F-SF	The PROMIS F-SF consists of seven items that measure both the experience of fatigue and the interference of fatigue on daily activities over the past week. The response scale is a 5-point Likert scale, ranging from 1 (never) to 5 (always). Total scores range from 7 to 35: a higher score indicating greater fatigue. The PROMIS F-SF showed acceptable reliability and validity and is suitable to measure fatigue across a diverse clinical population [49].	T0, T2, T3, T4, T5, T6
Muscle mass	Analyses of Computer Tomography	CT-analyses	CT images are routinely obtained in the oncological workup of lung and GI cancer patients, both pre- and postoperative. CT images obtained from whole-body PET/CT scans will be analyzed by a validated computer program AMUSE (acronym for Automatic MUscle and visceral/subcutaneous fat SEgmentor). The landmarks for the assessment of muscle mass will be the third lumbar vertebra (L3) and the fourth thoracic vertebra (Th4)	T0, T6
Protein and energy intake	48-h dietary recall		A 48-h dietary recall will be performed by trained interviewers, dietician researchers or graduate students, following a standard protocol. The intake of food items will be registered, including details of preparation methods, recipes, quantities and the size of portions, and the brands of products. The calculation of total energy intake and protein intake will be based on the database of ‘The Netherlands Nutrition Centre’. Protein intake will be calculated in gram per day. Energy intake will be calculated in kcal per day. Earlier studies suggested that a 48-h recall is superior to a single 24-h recall [50, 51].	T0, T2, T3, T5, T6
Physical performance	1) Hand grip strength 2) 30-s chair stand test 3) 2-min step test	1) - 2) 30SCST 3) TMST	1) Handgrip strength will be measured using the Jamar grip strength dynamometer as a measure of generalized muscle strength. The Jamar grip strength shows good reliability and can be used to measure changes in strength over time [52, 53]. The normative data of Dodds. et al. will be used as reference. [54] 2) The 30SCST will be used to measure functional lower extremity muscle functioning. The 30CST measures extremity strength in relation to demanding functional daily activities such as stair climbing [55]. The patient is seated in a chair and will be asked to complete as many full stands as possible in 30 s. The 30CST shows good reliability and criterion validity [56]. 3) The TMST will be used to measure exercise capacity [57]. The test requires the patients to march in place as fast as possible for 2 min while lifting the knees to a height midway between their patella and iliac crest when standing. Performance is defined as the number of steps completed in 2 min These performance tests are easy to perform at the patient’s home. The physical performance tests will be performed by trained (student) allied health professionals. Standard operating procedures will be used for all tests [58].	T0, T3, T5
Patient-specific activity limitations	Patient-specific Functional Scale	PSFS	The Dutch PSFS includes a list of 24 activities adapted from the activity list for patients with heart failure by Beurskens et al. and patients will be asked to indicate all activities that he or she was limited in performing within the previous week. [59] Patients have also the possibility to indicate any additional activities in the “other” section. The patient will be asked to prioritize their three most important activity limitations. Patients will then be asked to rate these activities on a numeric rating scale ranging from 0 to 10, where 0 indicates no limitations in performing the activity and 10 indicates that performing the activity is impossible. The PSFS is an appropriate measure for statistical comparisons in clinical research	T0, T2, T3, T4, T5, T6
Self-efficacy	General self-efficacy scale	GSES	The questionnaire contains 10 items with a 4-point response scale (1 = completely wrong, 4 = completely true). Total scores range from 10 to 40: a higher score indicating more self-efficacy. The GSES Is a valid tool to measure self-efficacy [60].	T0, T2, T3, T4, T5, T6
Participation in social roles and activities	CAT Dutch-Flemish Patient-Reported Outcome Measure Information System for participation	CAT PROMIS participation	The CAT PROMIS participation item bank including the item banks ‘Ability to participate in Social Roles and Activities’ and ‘Satisfaction with Social Roles and Activities’. The response scale is a 5-point Likert scale; a higher score indicates better participation. The CAT PROMIS item bank is a reliable and valid measurement of participation with limited administration time [61].	T0, T2, T3, T4, T5, T6
Generic Quality of Life	The 5-level EuroQol five-dimensional questionnaire	EQ-5D-5L	This EQ-5D-5L consists of five questions representing five health dimensions; mobility, self-care, usual activities, pain/discomfort and anxiety/depression. The EQ-5D-5L is added in order to carry out cost-effectiveness analysis. The EQ-5D-5L shows good psychometric properties and is suitable for economic evaluation studies in oncological patients [62].	T0, T2, T3, T4, T5, T6
Global perceived effect	Global Perceived Effect questionnaire	GPE	The Dutch version of the GPE will be used to measure the patient’s opinion of their recovery [63]. The questionnaire consists of two questions with a 7-point Likert scale. Patients will be asked to indicate to which extent they are recovered since the beginning of their recovery after surgery (1 = much better, 7 = much worse). In addition, patients will be asked how satisfied they are with their treatment since the beginning of their recovery after surgery (1 = very satisfied, 7 = very unsatisfied)	T0, T2, T3, T4, T5, T6
Hospital readmission	Medical Record Data		Unplanned *hospital readmissions* within 30 days after discharge will be reported	T3
Adverse event	Medical record data and self-reported		All adverse events with a direct or possible link to the OPRAH trial (e.g. AEs occurring during intervention-related activities) reported spontaneously by the subject or observed by the investigator or his staff will be recorded. This study will include patients undergoing oncological lung or GI surgery. These types of surgery are associated with a certain risk of postoperative complications. Our intervention starts after hospital discharge; therefore, all complications during hospitalization will not be reported as SAE. We expect that our, low-risk, post-discharge intervention will not have any negative influence on the occurrence complications after discharge. Therefore, all complications after discharge which are unmistakably caused by the surgery and/or medical treatment will not be reported as SAE. When there are doubts about the relation between the intervention and the occurrence of an SAE, these SAE’s will be discussed with the surgeons involved in this study	T1 t/m T6
***Other study parameters***				
Patient characteristics	Self-reported and Medical Record Data		Age (years), gender (male/female), body mass index (BMI), marital status (living together/alone), comorbidities, Mini-Mental State Examination (MMSE)*, The American Society of Anaesthesiologists Classification of physical health (ASA classification), tumor location (lung, upper GI, HPB, colorectal), stage of cancer (1–4), unintentional weight loss (% over de last 6 months), nutritional intake (% of nutritional requirements) *The MMSE will only be measured when there are doubts about the patient’s cognition. The MMSE is an instrument used for cognitive impairment in the elderly [64]. A low score on the MMSE corresponds to a low cognitive level. A score < 24 (out of 30) is usually considered deviant [65].	T0
Perioperative characteristics	Medical Record Data		Operation technique (open/laparoscopic), pre- or post-treatment with chemotherapy or radiotherapy, length of hospital stay (days)	T1
Self-management Skills	Self-Management Screening tool	SeMaS	The SeMaS is a short tool that can signal potential barriers for self-management that need to be addressed in the dialog with the patients and can facilitate personalized counselling. The following items of the SeMaS will be measured: level of education, experienced burden of disease, digital skills, locus of control, self-efficacy, social support, coping style, anxiety and depression. Based on the outcome factors of the SeMaS tool, the physiotherapist will give specific advice to the patients for the tailored intervention. The SeMaS is validated in patients with chronic condition in primary care [66]. However, this tool contains patient characteristics that are generally important in self-management and the questionnaire is not specific to a particular condition	T0
Treatment fidelity	Audio records		To investigate whether the intervention techniques used in the intervention (MI and SDM) have been implemented properly, some consultations with the physiotherapist and dietician will be audio-recorded. The audio records will be made with professional recording equipment and in no case with a telephone. The consultations will only be recorded if the patient has given his or her specific consent to record their consultations. Researchers trained in the use of MI and SDM will listen to these consultations to determine if the techniques have been used appropriately. The recordings will be made at multiple times during the study as a learning effect of the health professionals is expected. In addition, the number of consultations with the	T1 t/m T5
Treatment adherence	Intervention data		*Treatment adherence* will be measured to determine the extent to which the patients complied with the intervention. The following outcomes will be considered to determine treatment adherence: • Number of days of wearing the PAM sensor • Number of days of for which protein intake is recorded • Number of contact moments with the physiotherapist and dietician	T1 t/m T5
Process evaluation	Focus groups		A qualitative process evaluation will be performed to evaluate the implementation of the intervention in order to identify possible barriers and facilitators for further implementation. Focus groups will be conducted with patients from the intervention group, involved healthcare professionals and members of the research team to evaluate the intervention and implementation process. In addition, user adherence of the smartphone application and web application will be measured during the study	NA
Healthcare use	Self-reported and Medical Record Data		The number of consultations with a healthcare provider will be registered by asking the patients about their use of paramedic consultations in primary care and by screening the Medical Record data. This can be used to determine the difference in healthcare use and costs between the intervention and control group	

T0 = baseline (preoperative), T1 = at hospital discharge, T2 = 1 week after discharge, T3 = 4 weeks after hospital discharge, T4 = 8 weeks after hospital discharge, T5 = 12 weeks after hospital discharge, T6 = 6 months after hospital discharge, NA = not applicable

### Data collection

At baseline, the questionnaires will be sent by the researcher via the OnlinePROMS platform. After registration of the date of hospital discharge, the questionnaires will be automatically sent at 1 week, 4 weeks, 8 weeks, 12 weeks, and 6 months after hospital discharge. To improve the retention rate, patients will receive a reminder automatically after 2 and 5 days.

Standard operating procedures have been established for performing the physical measurements. All research assistants are trained to perform these measurements accurately and consistently. To ensure a higher follow-up rate, researchers have the flexibility to visit patients at home to perform the physical measurements. This approach aims to enhance convenience for participants and increase the likelihood of their continued participation in the study.

### Data management

Data obtained from the medical record and collected during baseline and follow-up measurements will be manually entered into Castor EDC. Castor EDC incorporates protection for data entry and validation to reduce data entry errors, and management features to facilitate audits and data quality assurance. Data from the online questionnaires will be saved at the OnlinePROMS database.

Both databases have been specifically developed to ensure the safeguarding of participant information in accordance with data protection regulations. Participants will be identified solely by a unique patient ID number, ensuring their anonymity. Trial-related documents will be securely stored and restricted to trial staff and authorized personnel only. Data will be anonymized promptly whenever feasible. All essential data that contains identifiable information will be retained for a period of 10 years, while anonymized digital data will be stored indefinitely. The Chief Investigator holds the role of the data manager, overseeing the management and security of the data.

Cleaned data sets will be made available to all Principal Investigators. These data sets will be securely stored on the research drive of Amsterdam UMC, VUmc location, and protected by passwords. Each Project Principal Investigator will have direct access to the data sets from their respective site, and access to data from other sites can be obtained upon request. To ensure confidentiality and privacy, any identifying participant information will be removed from the data sets if possible, when shared with project team members. Access to the full protocol, participant-level dataset and statistical code for non-commercial researchers outside the project team will be available from the corresponding author upon reasonable request.

### Statistical analysis

Missing data will be handled using longitudinal data analysis. The differences in course of recovery between groups, measured with the CAT PROMIS-PF, will be analyzed using linear mixed model analysis, with group as independent variable and the PROMIS-PF at all postoperative measurement points (*T*_1_–*T*_6_) as dependent variable, adjusting for baseline PROMIS-PF (*T*_0_). The primary analysis will be conducted based on the full analysis set according to the intention to treat method.

Descriptive statistics will be calculated for all parameters, include mean, median, standard deviation, standard error of the mean and the interquartile range. In addition, per-protocol analysis will be performed among all participants with sufficient protocol adherence (> 80%). Continuous secondary outcomes are analyzed using linear mixed model, with group as the independent variable and outcome at all postoperative measurement points as dependent variable, adjusting for baseline scores. Dichotomous outcomes are analyzed using generalized mixed model with the same multilevel structure. A mediation analyses will be performed on the longitudinal trial data to determine if the relationship between the intervention and the primary outcome (PROMIS-PF) can be explained by improvement of PA and protein intake.

Qualitative data analysis of the focus groups will be conducted following the steps of thematic analysis by two researchers.

### Dissemination policy

Research findings will be shared through publication in leading international peer-reviewed journals and through presentations at both national and international conferences. We are committed to disseminating the findings to all relevant stakeholders. In addition, a summary of the research findings will be sent to participants who have indicated that they would like to receive such information once the research findings are published. Standard authorship eligibility guidelines will be followed and professional writers will not be used.

## Discussion

The aim of this RCT is to evaluate the efficacy of a blended intervention to improve physical activity and protein intake on recovery of physical functioning in patients after gastrointestinal and lung cancer surgery. The OPRAH intervention, investigated in this RCT, aims to increase the patient’s self-management in physical recovery, by using a smartphone application, accelerometer and coaching by a physiotherapist and dietician to improve the patient’s level of physical activity and protein intake after hospital discharge. In addition, the OPRAH intervention aims to improve the collaboration between physiotherapy and dietetics in order to achieve an optimal synergy between nutrition and physical activity in patients after oncological GI and lung surgery. With this RCT, the short- and longer-term changes in physical functioning, physical activity, and protein intake will be determined with the hypothesis that a higher level of physical activity and protein intake will improve the recovery in physical functioning.

To improve recovery in physical functioning after oncological surgery, multiple studies have focused on prehabilitation, i.e. the process to enhance the patient’s functional capacity prior to major surgery, in order to enhance clinical outcomes and therefore reduce postoperative complications [67, 68]. However, surgery causes surgery-related muscle loss: muscle loss caused by increased catabolism due to surgical stress [7]. A recent study showed that more than 50% of the patients had surgery-related muscle loss [69]. Important risk factors that contribute to loss of muscle mass after oncological surgery are inactivity and malnutrition [9, 69]. To counteract the adverse effects of surgery, it is important that interventions also focus on encouraging physical activity and protein intake in the period after surgery.

This study has several strengths. First, the theoretical underpinnings of the intervention, by conducting a systematic review on the use of activity trackers and by using the Behavioral Change Wheel. Second, the tailored approach, by personalizing physical activity and protein goals for each patient and allowing the health professional to monitor the patient remotely. Third, an app that combines self-monitoring of physical activity and protein. This combination is unique and can facilitate collaboration between the physical therapist and dietitian. Fourth, a strength of the trial design is the use of blinded assessors.

A limitation of the study is that the intervention is only accessible for patients who are able to understand the Dutch language and have their own smartphone. However, based on our experiences with the feasibility studies, we have found that currently very few patients do not own a smartphone. In addition, a study is ongoing to make the Atris app more inclusive and therefore more accessible to patients with low health literacy or who do not speak the Dutch language. If our RCT shows effectiveness, the adapted app can be implemented in the OPRAH intervention.

To our knowledge, this is the first multicenter, assessor-blind RCT testing the effect of a blended intervention focused on improving physical activity and protein intake after discharge in patients who have undergone elective GI, HPB, or lung cancer surgery on the outcome recovery in physical functioning. The results of this research will reveal whether the OPRAH intervention, aiming to motivate patients and to assist health professionals to provide ongoing monitoring and support after hospital discharge, is an effective intervention to increase physical activity and protein intake and improve physical functioning recovery in these patients.

## Trial status

The most recent version of the protocol is version 3, updated on 5 July 2022. The recruitment started at 24 June 2022. Recruitment is expected to be completed by December 2023.

### Supplementary Information

**Additional file 1. **Intervention description and replication (TiDieR).

**Additional file 2. **Subscript screenshots figure 2.

**Additional file 3. **Mapping activities to MRC Framework.

**Additional file 4. **The behavioural change wheel.

## Data Availability

The datasets generated and/or analyzed are available from the corresponding author upon reasonable request.

## Abbreviations

BCT: Behavioral change technique
CAT: Computer Adaptive Testing
CONSORT: Consolidated Standards of Reporting Trials
EDC: Electronic Data Capture
ERAS: Early Recovery After Surgery
GI: Gastrointestinal
HPB: Hepato-pancreato-biliary
HIPEC: Hypertherme Intraperitoneale Chemotherapy
MET: Metabolic equivalents of energy expenditure
MI: Motivational interviewing
MMSE: Mini-Mental State Examination
MRC: Medical Research Council
OPRAH: Optimal Physical Recovery After Hospitalization
PROMIS: Patient-reported outcome measure information System
RCT: Randomized controlled trial
SDM: Shared decision making
