# Supplementary material for: The efficacy of a blended intervention to improve physical activity and protein intake for optimal physical recovery after oncological gastrointestinal and lung cancer surgery, the Optimal Physical Recovery After Hospitalization (OPRAH) trial: study protocol for a randomized controlled multicenter trial
Source: Trials. 2023 Nov 27;24:757. doi: 10.1186/s13063-023-07705-2 (PMC10680183; doi:10.1186/s13063-023-07705-2)
Supplement: Supplementary file 1 — Additional file 1. Intervention description and replication (TiDieR). [file 13063_2023_7705_MOESM1_ESM.pdf]

Supplementary File 1: intervention description and replication (TiDieR)

**Name**	OPRAH: A blended intervention, focused on self-monitoring, personal feedback and coaching regarding physical activity (PA) and protein intake, in patients with cancer planned for GI or lung surgery.
**Why**	Higher levels of PA in the perioperative period has a potential positive effect on recovery of physical functioning. However, stimulating PA in patients during hospitalization is challenging. The challenge to be physically active and resume daily activities can be even greater in the patient's own environment, where patients often experience barriers, such as physical symptoms, insecurity and lack of motivation or social support. Besides stimulating PA, it is also important to optimize the nutritional intake in the perioperative period, especially protein intake. Protein is the essential building block of muscle tissue and is needed in order to preserve muscle mass. Higher muscle mass has been shown to be an important factor in improving clinical outcomes in patients with cancer. Furthermore, protein is most effectively used for protein synthesis in combination with the stimulating effect of PA. However, previous studies found that many surgical patients were unable to meet their protein requirements after surgery and therefore additional support in protein intake is needed. Improved protein intake supports improvement in recovery of physical functioning. Self-monitoring is an effective and important behavior change technique, especially in combination with other coaching techniques e.g. goal setting, feedback on performance and social support.
**What (materials)**	The patients in the intervention group receive an ankle-worn PA monitor (PAM) and access to the corresponding application, Atris app, one week prior to surgery. The Atris app provides feedback on the number of active minutes per day. In addition, patients are able to self-monitor their protein intake by a simple self-registration tool. Patients have insight in their personal activity and protein goals, which are set in collaboration with their physiotherapist and dietician. The app provides non-specific rewards if their goals have been met. In addition, the physiotherapist and dietician will get notifications if the patients is not reaching their goal within a certain amount of time. These reminders can be tailored per patient. Via the app, patients are also able to chat with and receive personalized advice from the physiotherapist and dietician. Patients in the intervention group will also be given a leaflet containing information about physical activity and protein intake after surgery and additional information about the Atris app. Patients receive additional behavioural change support by a physiotherapist and dietician via telephone calls and chat messages. The coaching strategies of the physiotherapist and dietician will be based on the principles of Motivation Interviewing (MI).
**What (procedures)**	One week prior to surgery, patients receive the PAM and will receive access to and instructions about the Atris app to get familiarized with the application. Patients will be instructed by a flyer and a telephonic consultation with a researcher. Patients are asked to wear the PAM in a strap around the ankle, from at least one week prior to surgery until 3 months after surgery. During hospitalization, the treating physiotherapist and dietician guide the patients in the use of the app during their standard consultations. As discharge approaches, the patient will be supported by the physiotherapist and dietician using the shared decision making (SDM) process to set goals on active minutes and protein intake for after discharge. In case the dietician or physiotherapist is not able to meet the patient during hospitalization, the goals will be discussed by telephone in the first week after discharge. After discharge to home, patients are coached remotely (by telephone) by a physiotherapist and dietician about physical activity and protein intake. The goals and the degree of coaching will be tailored using a SDM process to the personal needs and preferences of the patient during the intervention period. To support patients in improving their self-management, MI techniques will be applied during the coaching sessions with the physiotherapist and dietician.
**Who provides**	The intervention will be provided by the physiotherapist and dietician

**How**	After randomization, the patients in the intervention group will receive the PAM, instructions to download the Atris app and informational leaflet by post. Patients will have access to the Atris app via their smartphone. During hospitalization or in the first week after discharge, goals will be set in collaboration with the physiotherapist and dietician. Patients will receive chat messages or telephone calls after discharge based on their personal needs and preferences.
**Where**	The intervention will be delivered remotely and participants will be able to access al the intervention elements at a location of their choice.
**When and how much**	Frequency of using the PAM, Atris app and informational leaflet will be at the discretion of the patients. Patients will receive chat messages and/or telephone calls by the physiotherapist and dietician based on their personal needs and preferences.
**Tailoring**	All patients will receive the same intervention materials. Personal goals on physical activity and protein intake will be tailored based on a SDM process with the physiotherapist and dietician. These goals can be adapted at any time and will be discussed via the chat or telephone calls. The number of phone calls and chat messages will be tailored based on the personal needs and preferences and if the physiotherapist or dietician assesses that there is added value for a contact moment, for example, if a patients has not achieved his goal for one week. The physiotherapist and dietician can tailor the notifications per patient (e.g. if a goal for 7 number of days is below 70%).
**How well**	In order to determine whether the intervention techniques used in the intervention (MI and SDM) have been implemented properly, some consultations with the physiotherapist and dietician will be recorded. The audio records will be made with professional recording equipment and in no case with a telephone. The consultations will only be recorded if the patients has given his or her specific consent to record their consultations. Researchers trained in the use of MI and SDM will listen to these consultation to determine if the techniques have been used appropriately. The recordings will be made at multiple times during the study as a learning effect of the health professionals is expected. In addition, other aspects of the treatment fidelity will be recorded:<br>• The number of consultations with the physiotherapist and dietician<br>• The delivery method of the intervention (telephone or chat).
