# Supplementary material for: The efficacy of a blended intervention to improve physical activity and protein intake for optimal physical recovery after oncological gastrointestinal and lung cancer surgery, the Optimal Physical Recovery After Hospitalization (OPRAH) trial: study protocol for a randomized controlled multicenter trial
Source: Trials. 2023 Nov 27;24:757. doi: 10.1186/s13063-023-07705-2 (PMC10680183; doi:10.1186/s13063-023-07705-2)
Supplement: Supplementary file 2 — Additional file 2. Subscript screenshots figure 2. [file 13063_2023_7705_MOESM2_ESM.pdf]

Supplementary File 2: Subscript screenshots figure 2

Three screenshots of the Atris app are presented in this figure. The left-hand screenshot presents the overview screen for the patient (dashboard). At the top is an overview of today's protein intake (Eiwit-inname). The protein intake is represented in stars (★), where 1 star represents approximately 5 grams of protein. In the circle diagram, the patient can see how many stars he or she has achieved in relation to his or her personal target. In this example the patients has achieved 11.5 out of 16 stars. The patient can also see how many stars he or she has achieved per day part(morning, afternoon, evening). If the patient clicks on "Eiwit-inname", the patient is led to the second screenshot. At the bottom of the first screenshot, the patient's personalized exercise goals (Beweegdoelen) are shown. In the circle diagram, the patient can see what percentage of their goal they have already reached today. In the second screenshot, the patient can record his or her protein intake per day. Using the arrows at the top, the patient can go back to previous days. At the top again is the circle diagram where the patient can see how many stars he or she has reached in relation to his or her personalized goal. By clicking on the '+' sign, the patient can enter his morning, afternoon and evening intake. If the patients clicks on the '+' sign, the patient is taken to the third screenshot. The third screenshot lists all the categories of intake that the patient can choose from. These are subdivided into bread, dairy, evening meal and snacks. See below for all the categories the patient can choose from.

Bread	Dairy	Evening meal	Snacks	Snacks
★★ Sandwich with savoury topping	★ Yoghurt or milk	★★ Quarter plate	★ Handful of nuts	½★ Cappuccino
★ Sandwich with sweet topping	★★★ Small bowl of Skyr or quark	★★★ Half plate	★ Egg	0★ Fruit or fruitjuice
★ Cracker with savoury topping	★★★ High protein yoghurt	★★★★★★ Full plate	★ Savoury snack	0★ Coffee, tea or soft drink
½★ Cracker with sweet topping	★ Cereals	★★ Meal soup	★★ Protein bar	½★ Sweet snack
★ Additional savoury topping (without sandwich)	★★★ Protein shake			
