# Supplementary material for: The efficacy of a blended intervention to improve physical activity and protein intake for optimal physical recovery after oncological gastrointestinal and lung cancer surgery, the Optimal Physical Recovery After Hospitalization (OPRAH) trial: study protocol for a randomized controlled multicenter trial
Source: Trials. 2023 Nov 27;24:757. doi: 10.1186/s13063-023-07705-2 (PMC10680183; doi:10.1186/s13063-023-07705-2)
Supplement: Supplementary file 3 — Additional file 3. Mapping activities to MRC Framework. [file 13063_2023_7705_MOESM3_ESM.pdf]

Supplementary file 3: Mapping activities to MRC Framework

				Described in this paper	
1	Development of the intervention			Methods	Results
1.1	Identifying evidence base	i.	Feasibility of self-monitoring in the target population	Leeuwerk et al., 2022 [26]	Leeuwerk et al., 2022 [26]
		ii.	Systematic review of interventions using activity trackers during and/or after an inpatient period	Leeuwerk et al., 2021 [21]	Leeuwerk et al., 2021 [21]
		iii.	Barriers and facilitators to the targeted behavior	Unpublished work	Unpublished work
1.2	Identifying/developing theory	i.	Literature review and meeting with expert group to decide on theory, behavioral change techniques and intervention strategies		✓
		ii.	Expert meetings with stakeholders to refine content and delivery mode		✓
1.3	Modelling process and outcomes	i.	Used behavioral change wheel to identify behavioral change techniques and mode of delivery that target the desired behavior.	✓	✓
		ii.	Evaluation with stakeholders to optimize practical effectiveness	✓	✓
2	Feasibility				
2.1	Testing the procedures for feasibility, compliance and intervention delivery	i.	Testing intervention on feasibility and compliance over 6-week post discharge intervention	Manuscript in preparation	Manuscript in preparation
		ii.	Evaluation with stakeholders to optimize practical effectiveness	Manuscript in preparation	Manuscript in preparation
2.2	Estimating recruitment and retention	i.	Test recruitment and retention from Amsterdam UMC	Manuscript in preparation	Manuscript in preparation
2.3	Determining sample size RCT	i.	Calculating sample size for the RCT with data from other comparable intervention trials	✓	✓

3	Evaluation				
3.1	Assessing effectiveness	i.	Set up a randomized controlled trial with recovery in patient reported physical functioning as primary outcome. Control group receives usual care.	✓	
3.2	Understanding change processes	i.<br>ii.<br>iii.	Assessing treatment fidelity by analyzing the recorded coaching sessions by the physiotherapist and dietician.<br>Assessing treatment adherence by recording the use of the app by the patient.<br>Mediation analysis to determine if the relationship between the intervention and the primary outcome can be explained by improvement of physical activity and protein intake	✓<br>✓	
3.3	Assessing cost-effectiveness	i.	Analyzing the cost-effectiveness of the intervention.		
4	Implementation				
4.1	Dissemination	i.	Developing implementation tools, conference presentations, peer review publications		
4.2	Surveillance and monitoring	i.	If the intervention is shown to be effective, implementation could be surveilled and monitored.		
4.3	Long term follow-up	i.	If the intervention is shown to be effective, process and outcome data could inform additional trials		
