# Supplementary material for: The efficacy of a blended intervention to improve physical activity and protein intake for optimal physical recovery after oncological gastrointestinal and lung cancer surgery, the Optimal Physical Recovery After Hospitalization (OPRAH) trial: study protocol for a randomized controlled multicenter trial
Source: Trials. 2023 Nov 27;24:757. doi: 10.1186/s13063-023-07705-2 (PMC10680183; doi:10.1186/s13063-023-07705-2)
Supplement: Supplementary file 4 — Additional file 4. The behavioural change wheel. [file 13063_2023_7705_MOESM4_ESM.pdf]

Supplementary File 4: The behavioural change wheel

COM-B components	What needs to change	Intervention functions		BCT's	Mode of delivery
**CAPABILITY**					
**Physical capability**	Have the physical skills to improve their level of physical activity (PA) and protein intake after surgery	Enablement	Reducing barriers to increase capability	1.2 Problem solving<br>2.2 Feedback on behavior<br>2.3 Self-monitoring of behavior<br>4.1 instructions on how to perform	Coaching by Healthcare Professional (HP)<br>Coaching by HP + Atris app<br>Atris app<br>Coaching by HP + Atris app
**Psychological capability**	Knowing how to improve their level of PA and protein intake after surgery	Education<br>Skills training	Educate about ways to be more physically active and about protein rich products<br>Train cognitive skills to be able to increase the level of PA and protein intake	2.2 Feedback on behavior<br>2.3 self-monitoring of behavior<br>4.1 instructions on how to perform	Coaching by HP + atris app<br>Atris app<br>Coaching by HP + Atris app
**OPPORTUNITY**					
**Physical opportunity**	Creating the opportunity to be physically active and to improve their protein intake in their own physical environment	Environmental restructuring	Provide cues and prompts to increase the level of PA	7.1 Prompts cues<br>12.5 Adding objects to the environment	Atris app<br>PAM accelerometer
**Social opportunity**	Creating access to support from a health professionals	Enablement	Enable patients to contact a physiotherapist or dietician	3.1 Social support	Coaching by HP + Atris app
**MOTIVATION**					
**Reflective motivation**	Patients need to understand and belief the benefits of increasing PA and protein intake on their recovery	Education, enablement	Inform about the benefits of increasing their level of PA and protein intake	1.7 review outcome goals<br>2.2 Feedback on behavior	Coaching by HP + Atris app<br>Coaching by HP + Atris app<br>Coaching by HP + Atris app

			Educate to create more positive beliefs about increasing PA and protein intake	5.1 Information about health consequences<br><br>2.3 self-monitoring of behavior	 <br>Atris app
	Patients need to have confidence in their ability to increase their level of PA and protein intake	Training, enablement<br><br><br>Persuasion, incentivisation	Train to set realistic goals.<br><br><br>Let the patient feel positive about increasing their level of PA and reaching their protein targets	1.1 Goal setting (behavior)<br>1.2 Problem Solving<br>1.4 Action planning<br>1.5 review behavioral goals<br>2.3 Self-monitoring of behaviour<br>3.1 Social support<br>8.7 Graded Tasks<br>10.3 non-specific reward<br>10.4 Social reward	Coaching by HP + Atris app<br><br>Coaching by HP<br>Coaching by HP + Atris app<br>Coaching by HP<br><br>Atris app<br><br>Coaching by HP + Atris app<br>Coaching by HP + Atris app<br>Atris app<br><br>Coaching by HP
**Automatic motivation**	Create established routines and habits to increase the level of PA and protein intake	Incentivisation, persuasion,	Reward patients if they continue increasing their level of PA and reach their protein targets	2.2 Feedback on behavior<br>2.3 self-monitoring on behavior<br>10.3 non-specific reward<br>10.4 Social reward	Coaching by HP + Atris app<br><br>Atris app<br><br>Atris app<br><br>Coaching by HP
